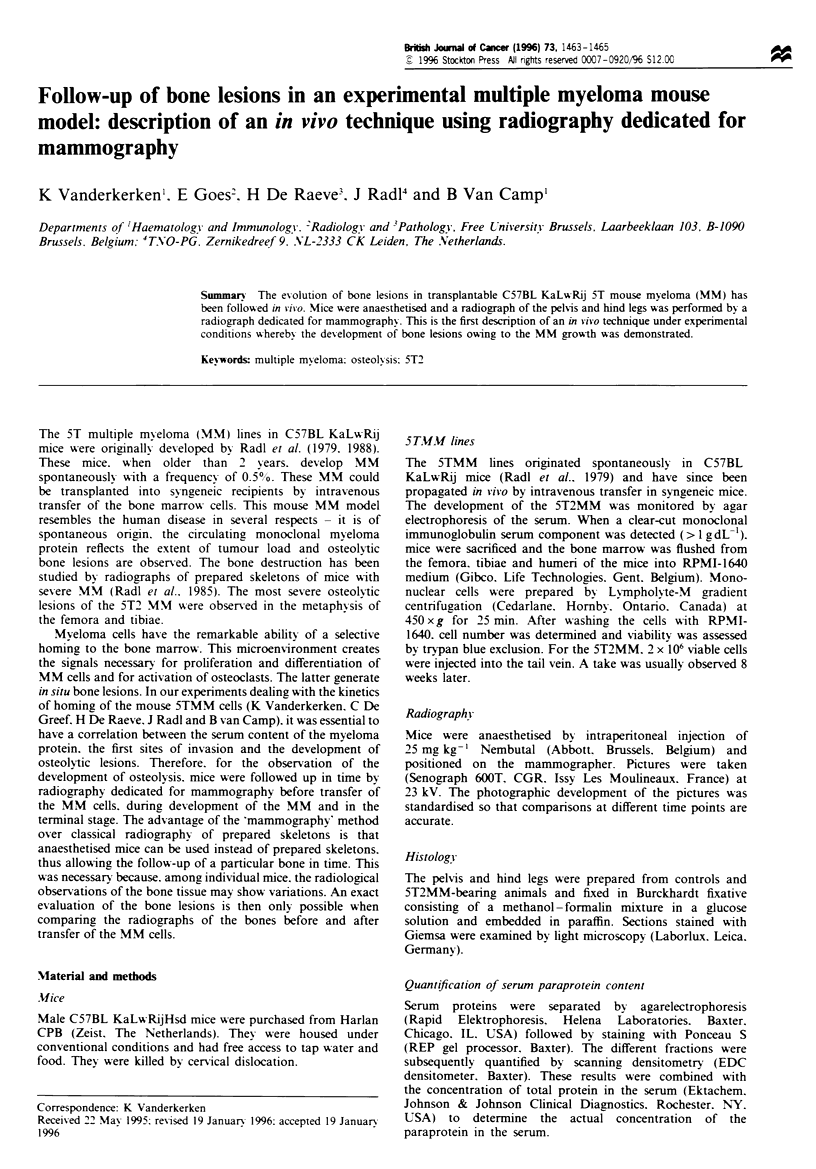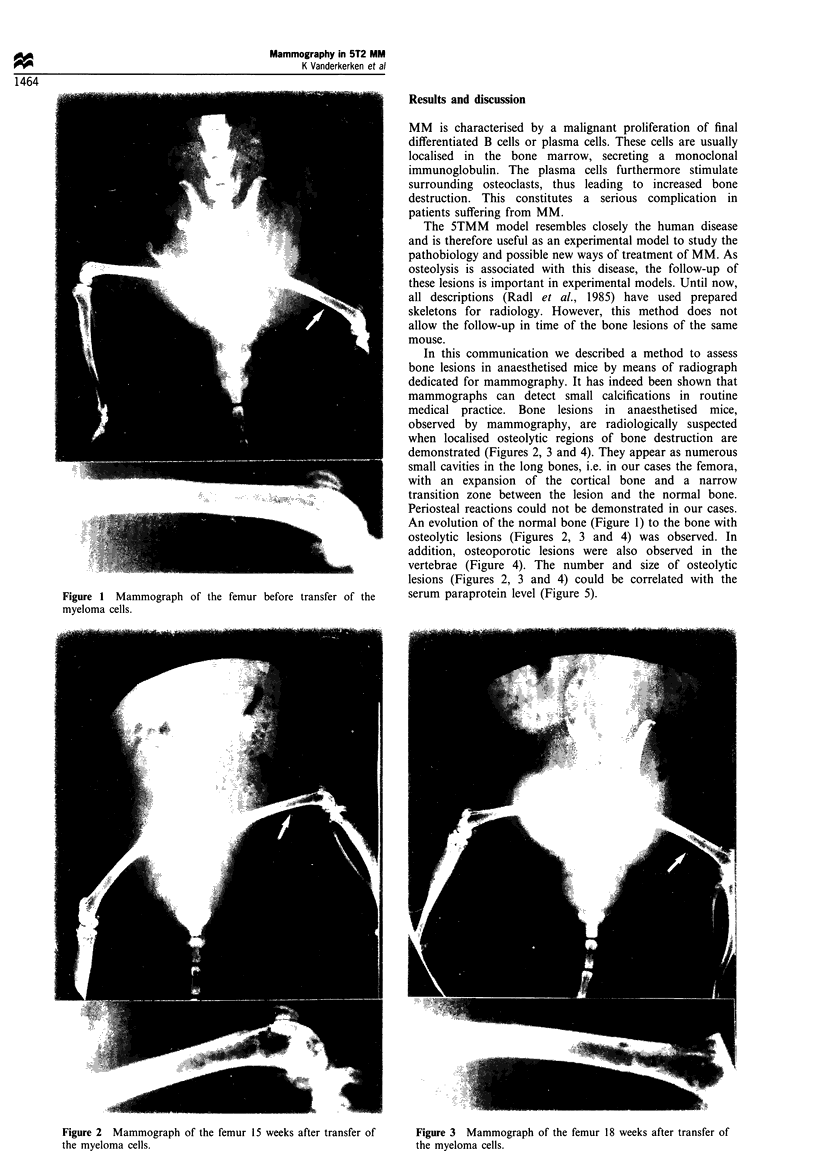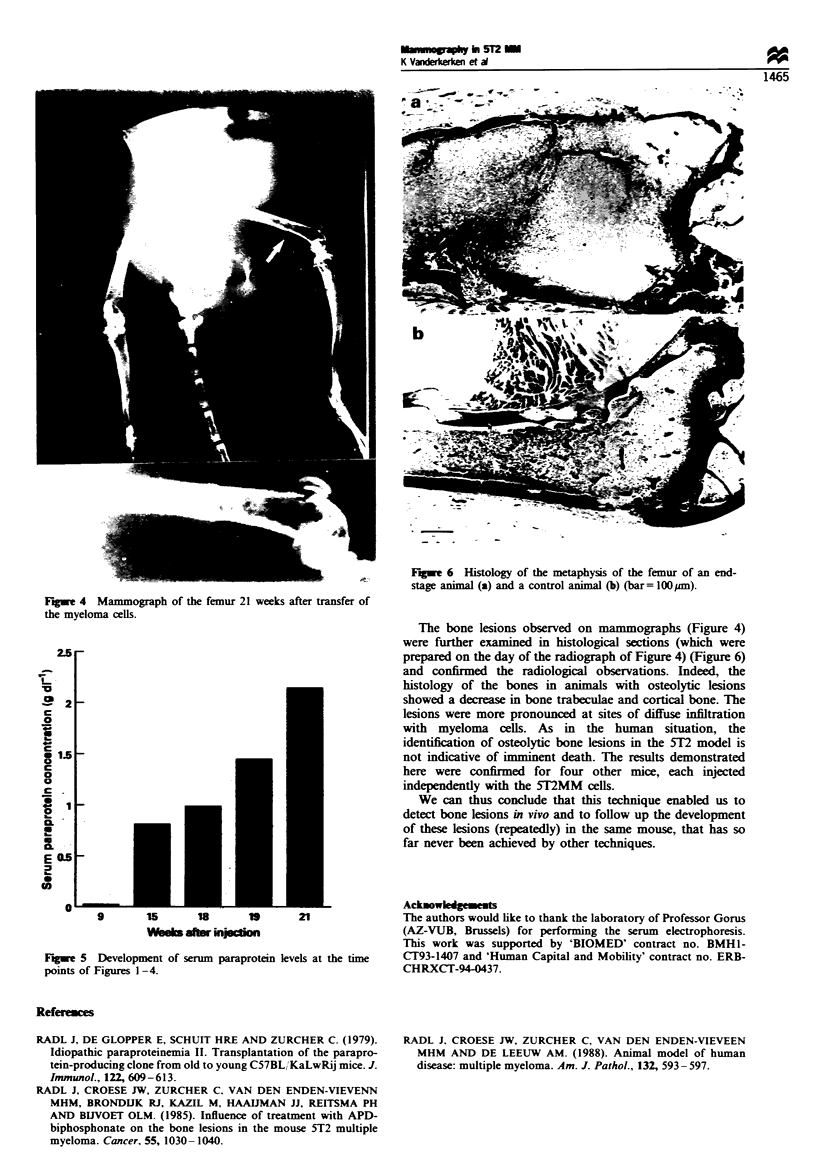# Follow-up of bone lesions in an experimental multiple myeloma mouse model: description of an in vivo technique using radiography dedicated for mammography.

**DOI:** 10.1038/bjc.1996.277

**Published:** 1996-06

**Authors:** K. Vanderkerken, E. Goes, H. De Raeve, J. Radl, B. Van Camp

**Affiliations:** Department of Haematology and Immunology, Free University Brussels, Belgium.

## Abstract

**Images:**


British Journal of Cancer (1996) 73, 1463-1465

© 1996 Stockton Press All rights reserved 0007-0920/96 $12.00

### Follow-up of bone lesions in an experimental multiple myeloma mouse model: description of an *in vivo* technique using radiography dedicated for mammography

K Vanderkerken[1], E Goes[2], H De Raeve[3], J Radl[4] and B Van Camp[1]

*Departments of [1]Haematology and Immunology, [2]Radiology and [3]Pathology, Free University Brussels, Laarbeeklaan 103, B-1090 Brussels, Belgium; [4]TNO-PG, Zernikedreef 9, NL-2333 CK Leiden, The Netherlands.*

**Summary** The evolution of bone lesions in transplantable C57BL KaLwRij 5T mouse myeloma (MM) has been followed *in vivo*. Mice were anaesthetised and a radiograph of the pelvis and hind legs was performed by a radiograph dedicated for mammography. This is the first description of an *in vivo* technique under experimental conditions whereby the development of bone lesions owing to the MM growth was demonstrated.

**Keywords:** multiple myeloma; osteolysis; 5T2

The 5T multiple myeloma (MM) lines in C57BL KaLwRij mice were originally developed by Radl *et al.* (1979, 1988). These mice, when older than 2 years, develop MM spontaneously with a frequency of 0.5%. These MM could be transplanted into syngeneic recipients by intravenous transfer of the bone marrow cells. This mouse MM model resembles the human disease in several respects – it is of spontaneous origin, the circulating monoclonal myeloma protein reflects the extent of tumour load and osteolytic bone lesions are observed. The bone destruction has been studied by radiographs of prepared skeletons of mice with severe MM (Radl *et al.*, 1985). The most severe osteolytic lesions of the 5T2 MM were observed in the metaphysis of the femora and tibiae.

Myeloma cells have the remarkable ability of a selective homing to the bone marrow. This microenvironment creates the signals necessary for proliferation and differentiation of MM cells and for activation of osteoclasts. The latter generate *in situ* bone lesions. In our experiments dealing with the kinetics of homing of the mouse 5TMM cells (K Vanderkerken, C De Greef, H De Raeve, J Radl and B van Camp), it was essential to have a correlation between the serum content of the myeloma protein, the first sites of invasion and the development of osteolytic lesions. Therefore, for the observation of the development of osteolysis, mice were followed up in time by radiography dedicated for mammography before transfer of the MM cells, during development of the MM and in the terminal stage. The advantage of the 'mammography' method over classical radiography of prepared skeletons is that anaesthetised mice can be used instead of prepared skeletons, thus allowing the follow-up of a particular bone in time. This was necessary because, among individual mice, the radiological observations of the bone tissue may show variations. An exact evaluation of the bone lesions is then only possible when comparing the radiographs of the bones before and after transfer of the MM cells.

### Material and methods

### *Mice*

Male C57BL KaLwRijHsd mice were purchased from Harlan CPB (Zeist, The Netherlands). They were housed under conventional conditions and had free access to tap water and food. They were killed by cervical dislocation.

Correspondence: K Vanderkerken

Received 22 May 1995; revised 19 January 1996; accepted 19 January 1996

### *5TMM lines*

The 5TMM lines originated spontaneously in C57BL KaLwRij mice (Radl *et al.*, 1979) and have since been propagated *in vivo* by intravenous transfer in syngeneic mice. The development of the 5T2MM was monitored by agar electrophoresis of the serum. When a clear-cut monoclonal immunoglobulin serum component was detected ($>1\ g\,dL^{-1}$), mice were sacrificed and the bone marrow was flushed from the femora, tibiae and humeri of the mice into RPMI-1640 medium (Gibco, Life Technologies, Gent, Belgium). Mononuclear cells were prepared by Lympholyte-M gradient centrifugation (Cedarlane, Hornby, Ontario, Canada) at $450 \times g$ for 25 min. After washing the cells with RPMI-1640, cell number was determined and viability was assessed by trypan blue exclusion. For the 5T2MM, $2 \times 10^6$ viable cells were injected into the tail vein. A take was usually observed 8 weeks later.

### *Radiography*

Mice were anaesthetised by intraperitoneal injection of $25\ mg\ kg^{-1}$ Nembutal (Abbott, Brussels, Belgium) and positioned on the mammographer. Pictures were taken (Senograph 600T, CGR, Issy Les Moulineaux, France) at 23 kV. The photographic development of the pictures was standardised so that comparisons at different time points are accurate.

### *Histology*

The pelvis and hind legs were prepared from controls and 5T2MM-bearing animals and fixed in Burckhardt fixative consisting of a methanol–formalin mixture in a glucose solution and embedded in paraffin. Sections stained with Giemsa were examined by light microscopy (Laborlux, Leica, Germany).

### *Quantification of serum paraprotein content*

Serum proteins were separated by agarelectrophoresis (Rapid Elektrophoresis, Helena Laboratories, Baxter, Chicago, IL, USA) followed by staining with Ponceau S (REP gel processor, Baxter). The different fractions were subsequently quantified by scanning densitometry (EDC densitometer, Baxter). These results were combined with the concentration of total protein in the serum (Ektachem, Johnson & Johnson Clinical Diagnostics, Rochester, NY, USA) to determine the actual concentration of the paraprotein in the serum.

Figure 1 Mammograph of the femur before transfer of the myeloma cells.

### Results and discussion

MM is characterised by a malignant proliferation of final differentiated B cells or plasma cells. These cells are usually localised in the bone marrow, secreting a monoclonal immunoglobulin. The plasma cells furthermore stimulate surrounding osteoclasts, thus leading to increased bone destruction. This constitutes a serious complication in patients suffering from MM.

The 5TMM model resembles closely the human disease and is therefore useful as an experimental model to study the pathobiology and possible new ways of treatment of MM. As osteolysis is associated with this disease, the follow-up of these lesions is important in experimental models. Until now, all descriptions (Radl *et al.*, 1985) have used prepared skeletons for radiology. However, this method does not allow the follow-up in time of the bone lesions of the same mouse.

In this communication we described a method to assess bone lesions in anaesthetised mice by means of radiograph dedicated for mammography. It has indeed been shown that mammographs can detect small calcifications in routine medical practice. Bone lesions in anaesthetised mice, observed by mammography, are radiologically suspected when localised osteolytic regions of bone destruction are demonstrated (Figures 2, 3 and 4). They appear as numerous small cavities in the long bones, i.e. in our cases the femora, with an expansion of the cortical bone and a narrow transition zone between the lesion and the normal bone. Periosteal reactions could not be demonstrated in our cases. An evolution of the normal bone (Figure 1) to the bone with osteolytic lesions (Figures 2, 3 and 4) was observed. In addition, osteoporotic lesions were also observed in the vertebrae (Figure 4). The number and size of osteolytic lesions (Figures 2, 3 and 4) could be correlated with the serum paraprotein level (Figure 5).

Figure 2 Mammograph of the femur 15 weeks after transfer of the myeloma cells.

Figure 3 Mammograph of the femur 18 weeks after transfer of the myeloma cells.

**Figure 4** Mammograph of the femur 21 weeks after transfer of the myeloma cells.

**Figure 5** Development of serum paraprotein levels at the time points of Figures 1–4.

**Figure 6** Histology of the metaphysis of the femur of an end-stage animal (**a**) and a control animal (**b**) (bar = 100 $\mu$m).

The bone lesions observed on mammographs (Figure 4) were further examined in histological sections (which were prepared on the day of the radiograph of Figure 4) (Figure 6) and confirmed the radiological observations. Indeed, the histology of the bones in animals with osteolytic lesions showed a decrease in bone trabeculae and cortical bone. The lesions were more pronounced at sites of diffuse infiltration with myeloma cells. As in the human situation, the identification of osteolytic bone lesions in the 5T2 model is not indicative of imminent death. The results demonstrated here were confirmed for four other mice, each injected independently with the 5T2MM cells.

We can thus conclude that this technique enabled us to detect bone lesions *in vivo* and to follow up the development of these lesions (repeatedly) in the same mouse, that has so far never been achieved by other techniques.

**Acknowledgements**
The authors would like to thank the laboratory of Professor Gorus (AZ-VUB, Brussels) for performing the serum electrophoresis. This work was supported by 'BIOMED' contract no. BMH1-CT93-1407 and 'Human Capital and Mobility' contract no. ERB-CHRXCT-94-0437.

### References

RADL J, DE GLOPPER E, SCHUIT HRE AND ZURCHER C. (1979). Idiopathic paraproteinemia II. Transplantation of the paraprotein-producing clone from old to young C57BL/KaLwRij mice. *J. Immunol.*, **122,** 609–613.

RADL J, CROESE JW, ZURCHER C, VAN DEN ENDEN-VIEVENN MHM, BRONDIJK RJ, KAZIL M, HAAIJMAN JJ, REITSMA PH AND BIJVOET OLM. (1985). Influence of treatment with APD-biphosphonate on the bone lesions in the mouse 5T2 multiple myeloma. *Cancer*, **55,** 1030–1040.

RADL J, CROESE JW, ZURCHER C, VAN DEN ENDEN-VIEVEEN MHM AND DE LEEUW AM. (1988). Animal model of human disease: multiple myeloma. *Am. J. Pathol.*, **132,** 593–597.